# Job Burnout among Safety Professionals: A Chinese Survey

**DOI:** 10.3390/ijerph18168343

**Published:** 2021-08-06

**Authors:** Bing Wang, Yuanjie Wang

**Affiliations:** 1 School of Resources and Safety Engineering, Central South University, Changsha 410083, China; safeboy@csu.edu.cn; 2 Safety & Security Theory Innovation and Promotion Center, Central South University, Changsha 410083, China; 3 Safety & Security Science and Emergency Management Center, Central South University, Changsha 410083, China

**Keywords:** safety professionals, job burnout, influencing factors, Chinese survey

## Abstract

As safety has been attracting the attention of all countries worldwide, the importance of safety professionals in safety management systems has been emphasized, which has consistently increased their workload. However, with the increase in work pressure, the income, social status, and social identity of safety professionals has not considerably improved, because of which the work motivation of safety professionals has reduced. Therefore, we aimed to identify the job burnout level (JBL) and its potential influencing factors among safety professionals in China. A total of 526 safety professionals from various industries participated. A univariate analysis of variance, independent sample t-test, bivariate correlation analysis, and multiple regression analysis were employed to analyze the situation of job burnout. An overwhelming majority of the safety professionals (98.3%) who participated in the questionnaire exhibited varying degrees of job burnout. The numbers of respondents with higher than normal emotional exhaustion (EE), lack of personal accomplishment (LPA), and depersonalization (DP) levels were 68, 474, and 381, respectively, accounting for 12.9%, 90.1%, and 72.4% of the total respondents, respectively. When different demographic characteristics were reviewed, the job burnout levels considerably varied. For example, male safety professionals (*n* = 434) exhibited higher levels of EE than female safety professionals (*n* = 92) (*p* = 0.025) because female safety professionals could release the dissatisfaction or stress they had encountered at work easily, but male safety professionals could not. Educational background had little effect on LPA (*p* > 0.05) and EE (*p* > 0.05), which indicated that job burnout was a general problem at all educational levels. The higher the age of respondents, the higher the level of LPA (*p* < 0.001). In addition to individual factors, work-related factors also had an impact on job burnout. For instance, monthly income had an impact on EE (*p* = 0.023) but had little impact on DP (*p* > 0.05). Furthermore, social, organizational, professional, and personal factors also had an impact on job burnout among safety professionals. Hence, to begin with, these aspects could be considered to alleviate the work pressure of safety professionals and reduce their job burnout levels.

## 1. Introduction

During the previous decades, the safety profession has experienced significant changes. With more and more safety regulations and social expectations on safety, the size and seniority of the safety profession in various organizations and industries has been expanding [1]. Safety professionals, who play a key role in influencing workplace safety, are the gatekeepers of safety [2]. The role of safety professionals in an organization is considered to assist the organization in the management of safety [3]. Over the past 30 years, they have become a growing force, extending safety services and improving people’s well-being. Today, safety professionals have been successfully integrated into the management structure of organizations [4]. They have grown into a professional team dedicated to safety, accident prevention, and emergency management. In addition, safety practitioners of governments worldwide are also committed to safety supervision, accident control, and safety education, and researchers in the field are performing safety experiments and research in various industries. Obviously, the growing importance of safety in the workplace has also led to a rising demand for safety professionals [5]. For example, since 2002, the Chinese government and safety researchers and practitioners have focused on the development of safety professionals (especially certified safety engineers) [6].

However, it is worth noting that different countries have different ways of managing safety professionals, and some countries have inadequate management mechanisms for them. Therefore, many safety professionals (especially grass-roots safety professionals) have heavy work tasks, high occupational pressure, prominent contradictions between power and responsibility, and low enthusiasm for work [7]. This has led to the emergence of a phenomenon of “job burnout” among safety professionals. This phenomenon has caused many problems. For example, in China, there have been several mass resignations of safety professionals in the past [8,9,10]. The survey results showed that these safety professionals generally felt greater mental stress, psychological burden, and easily complained about their work, hated performing their duties, and were not willing to complete their work. Moreover, there have been more extreme events in the United States. On 29 August and 24 September, two safety professionals at the Sago Mine in West Virginia shot themselves in their homes due to stress emanating from an investigation into an explosion that killed 12 miners in 2006 [11]. These cases all show that under the high pressure of safety management and safety responsibility, most safety professionals have problems such as being overburdened and lacking confidence in their career prospects. At the same time, compared with other departments, safety professionals often worked with large responsibilities, high requirements, and heavy tasks [7]. In the face of potential hazard management difficulties and poor working environments, sometimes even their own safety cannot be guaranteed among other problems, and thus safety professionals are more likely to appear job burnout [12]. Therefore, some safety professionals with low psychological endurance and excitable emotions are likely to have extreme behaviors in the above cases.

In the 1970s, clinical psychologist Freudenberger [13] and sociologist Maslach [14] first put forward the concept of job burnout. It is defined as a type of sub-health state of chronic stress, which is associated with limited resources, low abilities, and low energies and interests in long-term work [15]. This condition involves emotional exhaustion (EE), lack of personal accomplishment (LPA), and depersonalization (DP) [16,17]. These three indicators determine the job burnout level (JBL). In other words, JBL is measured by EE, LPA, and DP. More details are shown in Section 2.2 and Section 2.3. The formation process of job burnout expression is gradual, changing from quantity to quality [18]. At first, it appears in the form of stress. In the continuous development and change, when individuals gradually feel that their work energy is overdrawn, they gradually change into their attitude towards others and work. With the aggravation of the degree of stress, individual job burnout will eventually occur. Generally speaking, job burnout is an extreme negative reaction caused by an individual’s inability to cope with the work pressure calmly and effectively. It is the exhaustion of emotion, words and deeds, conduct, and work attitude under the action of long-term pressure [19].

In recent years, the seriousness of job burnout has attracted the attention of various countries and related international organizations. In 2018, job burnout was officially classified as an occupational phenomenon by the World Health Organization (WHO) in its latest revision of the International Classification of Diseases [20]. In addition, job burnout was recognized as an occupational disease in many countries [21]. Many studies have shown that there is a high rate of job burnout among teachers [22], health care professionals [23], police [24], and others. What these occupations have in common is that they all require a lot of emotional and energy from the person engaged in this work [12]. From this point of view, the safety profession also has similar characteristics [25], and the job burnout of safety professionals should paid attention to.

Job burnout exists among safety professionals and has become one of the major hazards affecting the stability of safety management systems [26]. It is a serious problem that can affect the physical and mental health of safety professionals, thereby reducing the individual’s performance and straining interpersonal relationships. However, existing research on job burnout mainly focuses on medical workers, educators, and public security officers [22,23,24]. In contrast, few studies have focused on the activities of occupational health and safety professionals [27]. Through a literature search, the authors found that there is limited research on the study of job burnout for safety professionals. Typically, Zhou [7] studied the occupational stress and job burnout of safety professionals (safety supervision staff) in China in the form of a questionnaire survey, which is very reliable and authentic. In addition, from a theoretical perspective, Chen et al. [12] elaborated the theoretical connotation of common job burnout and established a confirmatory game model to study job burnout for safety supervisors in Chinese coal mines. However, the limitations of their study lie in the fact that the respondents were from a single position (only from safety supervision staff) or a single industry (only from the coal industry), as well as the lack of comprehensive discussion on the causes of job burnout among safety professionals. Currently, no studies have focused on the overall situation of job burnout among safety professionals, and no targeted analyses of the causes of job burnout have been conducted. There is also a lack of research on strategies that can be implemented to reduce job burnout and improve productivity among safety professionals. In fact, safety professionals are facing a grim situation that results from not only society or the profession itself, but also from their organizations and themselves.

The general factors that cause job burnout include individual factors and work-related factors [28,29]. The importance of these factors in safety professionals’ job burnout is different. In terms of individual factors, previous studies have confirmed that gender, age, and educational background can affect the job burnout level [30,31,32]. For example, older safety professionals and married safety professionals reported higher LPA levels, and the EE of male safety professionals was more severe than that of female safety professionals. In terms of the work-related factors, daily working hours, income, and years of employment are important factors that affect job burnout [17,33,34,35]. For example, the longer safety professionals worked a week, the more depersonalized they became, and the higher their JBL was. The higher the monthly income, the higher the LPA levels. Moreover, the degree of influence of other factors on job burnout could be different among safety professionals. Whether these factors affect job burnout remains unclear, and these assumptions have not been confirmed. If these problems about the job burnout of safety professionals cannot be properly solved, it will inevitably affect their own health and personal work performance, and even affect the construction of safety management teams and the healthy and harmonious development of the whole society [7].

Therefore, the purpose of this study was to determine whether these individual factors and work-related factors affect the JBL among safety professionals and to explore the important causes of job burnout among safety professionals from four dimensions of negative perception—social factors, occupational factors, organizational factors, and personal factors. The specific objectives are as follows: (a) to analyze the level of job burnout among safety professionals with different characteristics; (b) to identify the factors that influence burnout among safety professionals; and (c) to propose policy recommendations to address these issues.

## 2. Materials and Methods

### 2.1. Study Population

A study on job burnout among Chinese safety professionals was conducted from July 2020 to November 2020. Safety professionals distributed across various industries in China were included. They were randomly selected to participate in the survey. In this study, in total, 550 questionnaires were sent to government departments, public institutions, foreign-funded or joint ventures, state-owned enterprises, private enterprises, etc. A total of 526 safety professionals completed the questionnaire survey; the professionals belonged to coal mining, metal and non-metallic mining, chemical industry, construction, road transportation, metal smelting, fast-moving consumer goods, safety consulting, government supervision, and other industries. The response rate was 95.6% (526/550).

### 2.2. Measurement Tools

As for the measurement tools of job burnout, we find that Maslach Burnout Inventory (MBI) is relatively common at present. Its application in different regions, fields, and cultures shows practicability and objectivity [36]. The MBI has three versions: the MBI-human Service Survey (MBI-SS), MBI-Educators Survey (MBI-ES), and MBI-General Survey (MBI-GS) [37,38]. In this study, the job burnout of safety professionals was measured using a Chinese version of MBI-GS, which was translated and revised by Chaoping Li [39,40,41]. The Chinese version of the MBI-GS uses 16 questions to measure JBL, and the revised measurement scale is completely consistent with the original structure of the MBI-GS, which indicates that the MBI is also effective in China. Meanwhile, many verifications have been conducted, indicating that MBI has good reliability and validity in China as well [39].

The MBI-GS used in this study included three indicators: EE, LPA, and DP. There was a total of 16 items and the values of Cronbach’s α were 0.901. The 3 subscale items (EE, LPA, and DP) were 5, 6, and 5, and the values of Cronbach’s α were 0.923, 0.894, and 0.940, respectively. The results of the reliability test demonstrated that each Cronbach’s α was higher than 0.8, and the overall reliability was high. Each item was scored from 0 to 6 on the self-reported frequency of feelings the respondents addressed. Based on the sound project scoring criteria of other industries [42,43,44], this paper applies it to the study of job burnout of safety professionals, and cites the following project scoring criteria as the research basis.

EE was composed of five items, with a total score range of 0 to 35. Scores greater than or equal to 27 (scores ≥ 27) were considered to be job burnout in terms of EE, whereas scores less than 27 (scores < 27) were considered normal (no burnout). LPA included six items for a total score range of 0 to 42. Scores less than or equal to 24 (scores ≤ 24) were considered to be job burnout in terms of LPA, whereas scores greater than 24 (scores > 24) were considered normal (no burnout). DP included five items for a total score range of 0 to 35. Scores greater than or equal to 8 (scores ≥ 8) were considered to be job burnout in terms of DP, whereas scores less than 8 (scores < 8) were considered normal (no burnout). The status of the three indicators would determine the final JBL of the respondents (see Section 2.3 for an introduction to JBL).

In addition, to explore the causes of job burnout among safety professionals, in this study, based on the theoretical research on job burnout and the development status of safety professionals, we compiled 14 multiple-choice questions (MCQs) to investigate and study the negative perception of jobs among safety professionals. We used MCQ dichotomy data, quantified the four dimensions of negative perception, and analyzed the correlation between the scores for the four negative perception and job burnout. In addition, a regression model was developed using ordered dummy variables from the demographic data to analyze the effects on job burnout. See Section 3.3 for more details.

### 2.3. Variables

The independent variables were the individual factors and work-related factors that may have influenced job burnout. Individual factors included (a) gender, (b) age, (c) educational background, and (d) marital status. Work-related factors included (e) monthly income (RMB), (f) weekly work hours, (g) work experience (years), (h) position level, (i) if the individual was a certified safety engineer (CSE), and (j) if the individual held an administrative position.

JBL was the dependent variable and was measured using MBI-GS from EE, LPA, and DP. Referring to Li’s judgment method [24], JBL was divided into four levels. If all three indicators (EE, LPA, and DP) were normal, JBL was “no burnout.” If one of the indicators was abnormal, JBL was “mild-level burnout.” If two of the indicators were abnormal, JBL was “middle-level burnout.” If all three indicators were abnormal, JBL was “high-level burnout”.

### 2.4. Statistical Analysis

After collecting and sorting all the questionnaire data, SPSS 22.0 (IBM, Armonk, NY, USA) was used to analyze and process the data. Enumerated data was expressed as frequencies and percentages, and the measurement data was expressed as mean and standard deviation. For analysis of the effect of a single factor on the dependent variable, univariate analysis of variance, independent sample *t*-test, and bivariate correlation analysis were employed, according to the attribute characteristics of independent variables. Multiple regression analysis was employed to analyze the comprehensive influence of multiple factors on the dependent variables. Variables were measured using the 95% confidence interval (*p* < 0.05), which was considered statistically significant.

## 3. Results

### 3.1. General Characteristics of Study Population

The general characteristics of the subjects are presented in Table 1. In total, 526 subjects were enrolled, which included 434 males and 92 females, most of whom were younger than 40 years (66.2%). One hundred and ninety-five respondents were aged 31–40, accounting for 37.1% of the total respondents. The number of married people was the highest at 371, accounting for 70.5% of all respondents. More than half of the safety professionals had an undergraduate qualification (53.6%). Less than half of the safety professionals were grass-roots managers (41.8%), 48.5% were certified safety engineers, and 39.7% held administrative positions. Respondents had relatively average work experiences (below 20 years) and monthly salaries (RMB). The numbers of safety professionals with years of work experience below 5, 6–10, and 11–20 were 139, 143, and 143, respectively, accounting for 32.1%, 27.2%, and 27.2% of all respondents, respectively. The number of safety professionals with monthly salaries below 5000 (RMB), 5000–7000, 7000–10,000, and above 10,000 were 125 (23.7%), 148 (28.1%), 117 (22.1%), and 137 (26.1%), respectively. The work hours for 341 safety professionals, accounting for 64.8% of the total respondents, were concentrated in the range of 40–60 h per week (see Table 1).

### 3.2. The Difference of Job Burnout Level When Different Demographic Characteristics Were Reviewed

According to the statistical results of the JBL of safety professionals, the number of respondents who experienced no burnout was the lowest at only nine, accounting for 1.7% of all respondents. One hundred and sixty-one respondents experienced mild-level burnout, which accounted for 30.6% of all respondents. Safety professionals who experienced middle-level burnout were in the majority, with 306 respondents, accounting for 58.2% of all respondents. Fifty respondents experienced high-level burnout, accounting for 9.5% of the total respondents (see Table 1).

In addition, the results of the statistical analysis revealed that the mean EE score was 16.5, which indicated normal level. However, the mean scores of LPA and DP were 12.8 and 13.8, respectively, indicating burnout level. According to the evaluation criteria for job burnout [24], among the 526 safety professionals, 68 experienced EE burnout, accounting for 12.9% of the total number. There were 474 and 381 respondents with LPA burnout and DP burnout, respectively, accounting for 90.1% and 72.4% of all respondents (see Table 2).

Because the demographic data was divided into three types—two-level dummy variables, multi-level unordered dummy variables, and ordered dummy variables—different methods were adopted in this part of the analysis according to the characteristics of the three types of variables. The independent sample t-test was applied to two-level dummy variables, a one-way analysis of variance (ANOVA) was applied to multi-level unordered dummy variables, and a Pearson correlation analysis was applied to the ordered dummy variables. The outcome of these analyses revealed that EE, LPA, and DP were statistically correlated with JBL (see Table 3). The analysis results were as follows:

1. From the perspective of gender difference, no statistically significant difference in LPA (*p* > 0.05) and JBL (*p* > 0.05) was observed between the two groups of respondents. However, the EE (*p* = 0.025) and DP (*p* = 0.033) for males were higher than those for females. Overall, the JBLs of male and female safety professionals were very similar.
2. In terms of age, the higher the age of the respondents, the lower the scores of LPA (*p* < 0.001) and DP (*p* < 0.001). No significant correlation was observed between EE (*p* > 0.05) and age. The Pearson correlation analysis revealed that age and JBL were significantly correlated, and the Pearson correlation coefficient was −0.086 (*p* = 0.048); thus, the older the respondents, the lower their burnout levels.
3. From the perspective of educational background, no significant correlation between educational background of the respondents and the EE (*p* > 0.05), LPA (*p* > 0.05), DP (*p* > 0.05), JBL (*p* > 0.05) was observed. It can be observed that the JBL among safety professionals was not related to the educational background.
4. With regard to the marital status of the respondents, the one-way ANOVA indicated that there were significant differences between the LPA (*p* = 0.002) and DP (*p* < 0.001) of the respondents with different marital statuses. The LPA and DP scores of unmarried people were significantly higher than those of married people. In addition, there was no significant difference between different marital status in EE (*p* > 0.05) and JBL (*p* > 0.05).
5. In terms of the monthly income of the respondents, there was no significant correlation between EE (*p* > 0.05), DP (*p* > 0.05), and JBL (*p* > 0.05) and monthly income. However, the higher the monthly income of the respondents, the lower the LPA scores (*p* = 0.023).
6. With regard to the weekly work hours of the respondents, there was no significant correlation between LPA (*p* > 0.05) and weekly work hours. However, the longer the weekly work hours, the higher the scores of EE (*p* < 0.001), DP (*p* < 0.001), and JBL (*p* = 0.011). The Pearson correlation coefficient between weekly work hours and JBL was 0.111.
7. In terms of work experience, no significant correlation was observed between EE (*p* > 0.05) and work experience. However, the longer the working years, the lower the scores of LPA (*p* < 0.001), DP (*p* < 0.001), and JBL (*p* = 0.045). The Pearson correlation coefficient between work experience and JBL was −0.087.
8. From the perspective of position level, no significant correlation in EE (*p* > 0.05) and JBL (*p* > 0.05) and the position level of the respondents was observed. However, the higher the position level, the lower the LPA score (*p* = 0.004) and DP (*p* = 0.044).
9. Based on whether safety professionals held administrative positions, an independent sample t-test was conducted on the three subscales of job burnout of the two types of respondents. The results revealed that the respondents who did not hold administrative positions had significantly higher LPA scores (*p* = 0.028) than those who held administrative positions. There was no significant difference between the two groups in terms of EE (*p* > 0.05), DP (*p* > 0.05), and JBL (*p* > 0.05).
10. Based on whether safety professionals were CSEs, an independent sample t-test was conducted on the three subscales of job burnout of the two types of respondents. The results indicated that there was no significant difference between the two groups in terms of EE (*p* > 0.05), LPA (*p* > 0.05), and DP (*p* > 0.05). However, the JBL (*p* = 0.034) of the respondents who were CSEs was higher than that of respondents who were not.

### 3.3. Analysis of the Effects of Negative Perception on Job Burnout

The choices available for the four negative perception dimensions were analyzed; the proportions of each choice are shown in Table 4. In terms of the proportion of choices, the majority of choices accounted for more than 50%, indicating that the respondents held more negative views on their safety work. After performing a reliability test of the four negative perception scores, Cronbach’s α coefficient was found to be 0.641, indicating that the overall reliability was acceptable. Further statistical analysis of the scores of the four negative perception dimensions revealed that the scores of the negative perception of social factors and occupational factors were higher, which indicated that the respondents were more dissatisfied with the social environment and the occupational conditions of safety professionals. In contrast, the scores of negative perceptions of organizational factors and personal factors were mainly concentrated in the lower segment, indicating that the respondents’ perception of these two dimensions of negative factors was relatively low (see Table 5).

In terms of the relationship between negative perception factors and job burnout, the respondents were grouped according to the choice of each option. The grouping criterion was whether a factor of the dimension of negative perception was selected (that is, an option was selected), and the scores of EE, LPA, DP, and JBL were considered as dependent variables. An independent sample t-test and Pearson’s correlation analysis were conducted, and the test and analysis results are shown in Table 6. According to the results, the impact of negative perception factors on job burnout can be summarized as follows:

1. In terms of the negative perception of social factors, respondents who selected Q1-1 had higher LPA (*p* < 0.01) and DP (*p* < 0.01) scores than those who did not; respondents who selected Q1-2 had higher DP (*p* < 0.05) scores than those who did not. A significant positive correlation was observed between the negative perception of social factors (Q1) and the EE (*p* < 0.01) and DP (*p* < 0.05) scores; hence, the higher the scores of the negative perception of social factors, the higher the scores of EE and DP (see Table 6).
2. In terms of the negative perception of occupational factors, respondents who selected Q2-1 had higher EE (*p* < 0.01), DP (*p* < 0.05), and JBL (*p* < 0.05) scores than those who did not. Respondents who selected Q2-2 had higher EE (*p* < 0.01) and JBL (*p* < 0.05) scores than those who did not. Respondents who selected Q2-3 had higher DP (*p* < 0.05) scores than those who did not. Respondents who selected Q2-4 had higher EE (*p* < 0.01) scores than those who did not. A significant positive correlation was observed between the negative perception of occupational factors (Q2) and EE (*p* < 0.01), DP (*p* < 0.01), and JBL (*p* < 0.01). In other words, the higher the scores of the negative perception of occupational factors, the higher the scores of EE, DP, and JBL (see Table 6).
3. In terms of the negative perception of organizational factors, respondents who selected Q3-1 had higher EE (*p* < 0.01), LPA (*p* < 0.01), DP (*p* < 0.01), and JBL (*p* < 0.01) scores than those who did not. Respondents who selected Q3-4 had higher EE (*p* < 0.05) scores than those who did not. A significant positive correlation was observed between the negative perception of organizational factors (Q3) and EE (*p* < 0.01), DP (*p* < 0.01), and JBL (*p* < 0.01) scores; in other words, the higher the scores of the negative perception of organizational factors, the higher the scores of EE, DP, and JBL (see Table 6).
4. In terms of the negative perception of personal factors, respondents who selected Q4-1 had lower DP (*p* < 0.05) scores than those who did not. Respondents who selected Q4-2 had higher EE (*p* < 0.01), LPA (*p* < 0.01), DP (*p* < 0.01), and JBL (*p* < 0.05) scores than those who did not. Respondents who selected Q4-3 had higher LPA (*p* < 0.05) scores than those who did not. The negative perception of personal factors (Q4) was significantly positively correlated with the LPA (*p* < 0.01) and DP (*p* < 0.05) scores; thus, the higher the scores of the negative perception of personal factors, the higher the scores of LPA and DP (see Table 6).

### 3.4. Regression Analysis of Job Burnout

In combination with the above analysis results, four regression models were developed using the negative perception scores of the four dimensions as independent variables and the scores of the three subscales of job burnout (EE, LPA, and DP) and JBL as dependent variables. To select the four independent variables, the model incorporated demographic variables significantly related to the corresponding dependent variables into the model. For example, both gender and weekly work hours had an impact on EE; therefore, the two variables were included in the regression model of EE and analyzed in combination with the four independent variables of negative perception. The analysis results are as follows:

1. The previous analysis revealed that both gender and weekly work hours had an impact on EE scores; thus, the regression model of EE took gender, weekly work hours, and negative perception scores in four dimensions as independent variables. It was found that the results of model variance analysis were significant (*p* < 0.05). The results showed that the weekly work hours (*p* < 0.001), the negative perception of occupational factors (Q2, *p* = 0.003), and the negative perception of organizational factors (Q3, *p* = 0.047) all had significant positive effects on EE. In other words, the longer the weekly work hours, the higher the levels of Q2 and Q3, and the higher the EE levels of safety professionals (see Table 7).
2. The analysis results showed that the five variables—age, work experience (years), position level, whether the respondent held an administrative position, and monthly income (RMB)—all affected LPA. Therefore, in the regression model of LPA, these five variables and the negative perception scores of the four dimensions were taken as independent variables. The results of model variance analysis were significant (*p* < 0.05). The results showed that only age (*p* = 0.037) and negative perception of personal factors (Q4, *p* < 0.001) had significant effects on LPA. Among them, age had a negative impact on personal achievement; that is, the older the respondent, the lower the LPA scores. Q4 had a positive impact on personal achievement. In other words, the higher the scores of Q4, the higher the LPA scores (see Table 8).
3. The previous analysis showed that gender, age, work experience (years), position level, and weekly work hours had an impact on DP; thus, in the regression model of DP, these five variables and the negative perception scores of the four dimensions were taken as independent variables. The results of model variance analysis were significant (*p* < 0.05). The results showed that gender (*p* = 0.042), weekly work hours (*p* = 0.004), and negative perception of organizational factors (Q3, *p* < 0.001) all had significant effects on DP. Male safety professionals scored higher than female safety professionals. Weekly work hours and Q3 had a positive influence on the DP scores. The longer the weekly work hours, the higher the DP scores. The higher the scores of Q3, the higher the DP scores (see Table 9).
4. The analysis results showed that the four variables—age, work experience (years), if the individual was a CSE, and weekly work hours—had an effect on JBL. Therefore, in the regression model of OBL, these four variables and the negative perception scores of the four dimensions were taken as independent variables. The results of model variance analysis were significant (*p* < 0.05). The results showed that only the variable of whether the respondents were CSEs (*p* = 0.019) had a significant impact on the JBL; in other words, the JBLs of the respondents who were CSEs were higher than that of respondents who were not CSEs (see Table 10).

## 4. Discussion

According to the above analysis results, the effects of the 10 demographic variables and 4 negative perception factors are presented in Table 11. The influence of these factors on job burnout of safety professionals is discussed and summarized as follows:

In total, seven factors had a significant effect on EE (see Table 11). Male safety professionals exhibited higher EE levels than their female counterparts (see Table 3). Women have unique physiological characteristics and social attributes; thus, they can easily find an outlet for stress. Whether by talking to others or regulating their emotion, female safety professionals can release the dissatisfaction or stress they have encountered at work, potentially lowering their EE levels. In contrast, male safety professionals are more inclined to internal digestion and are reluctant to seek external support [45]. If there is no outlet for negative psychological states, the EE levels will only increase and not easily decrease [46]. In addition, as weekly work hours increase, the EE levels of all respondents also increase (see Table 3 and Table 7). The legal weekly work hours in China are 40 h, with an average of eight hours per day. However, because of various circumstances, some safety professionals have to work more than the legal working hours per week [7]. Constant overtime and heavy workloads drain their energy and may result in higher EE levels. In terms of negative perception factors, safety professionals with relatively high EE levels believe that their work pressure is high, their interpersonal relationships are complex, their working objects vary greatly, their work environment is not good, their work pay is low, and their personality is not suitable for working in the safety industry (see Table 6 and Table 7).

A total of ten factors significantly affected LPA (see Table 11). The reverse scoring method was adopted for LPA, that is, the lower the scores, the higher the LPA levels. In terms of personal factors, two items had a significant effect on the LPA levels (see Table 3 and Table 8). Young safety professionals (under 30 years old) are just starting their career and are full of enthusiasm and motivation for work. At the same time, most of them are not married and do not have the burden of family. They devote more energy to their safety work and feel satisfied and fulfilled by their achievements. These reasons may lead to lower LPA levels. However, as the age increases, safety professionals become accustomed to their type of work. In addition, safety professionals over 30 years old also are likely to take care of their children’s education and support their parents while working [7]. They cannot easily feel a sense of achievement from their work. These factors undoubtedly have a considerable impact on their achievement at work, leading to an increase in LPA levels. LPA levels for safety professionals who are 41 years and older are significantly higher than those for safety professionals under 41 years of age. LPA levels of unmarried safety professionals were significantly lower than those of married safety professionals, and the LPA levels of married people were not significantly different from those of divorced or widowed people. In terms of work-related factors, four items had a significant effect on LPA levels (see Table 3). The length of service of safety professionals is mostly spread over the periods of 6–10 years and 11–20 years. From the viewpoint of career development of safety professionals, these two stages are in the middle—the frustration period and the stop period. Therefore, their LPA levels are not different from each other. They exhibit resistance and burnout to work and lack a personal sense of achievement. Safety professionals with less than 5 years of work experience may be at the development stage and growth stage when they are full of enthusiasm for their work; thus, their LPA levels are low. However, safety professionals with 21 years or more of work experience have entered the stage of career decline. Although they experience less pressure in life, they are less motivated to work and achieve less during work, potentially leading to the highest LPA levels. In terms of monthly income and position level, the analysis results revealed that the LPA levels of senior managers and safety professionals with monthly incomes of more than 10,000 are the highest in the corresponding groups. This is a point of great concern for researchers. In general, as people move up the career ladder and earn more money, they have more resources at their disposal and use, and their sense of accomplishment increases. Among safety professionals, however, the opposite is true. With the increase in monthly income, the LPA levels increase. This shows that the higher income must be accompanied by high work pressure. The work pressure constantly drains their sense of personal accomplishment, making them lose their joy in work and forget the value of work. As safety professionals move up the ranks, they become more responsible and busier. In a higher position, it is difficult for them to get the satisfaction and sense of achievement that they had at the beginning of their careers. The responsibility and pressure they bear make it difficult for them to get more happiness from work. Simultaneously, they also feel that their value is not reflected in their work. These factors lead to an increase in their LPA levels. In addition, safety professionals who hold administrative positions need to spend time and energy in dealing with administrative affairs while completing safety-related work. They are under considerable pressure for promotion and generally lack a high sense of achievement. Therefore, their LPA levels are also relatively high. In terms of negative perception factors, safety professionals with relatively high LPA levels believe that their social status is not high, their work environment is not good, their personality is not suitable for the safety industry, personal awareness of the safety industry is insufficient, and it is difficult to achieve their self-value (see Table 6 and Table 8).

Twelve factors significantly affected DP (see Table 11). In terms of personal factors, three items considerably affected LPA levels (see Table 3 and Table 9). Analysis results show that male safety professionals exhibit higher DP levels than their female counterparts. In general, in the safety industry, male safety professionals generally have more work pressure than female ones. Because men have fewer ways of regulating their emotions than women, men are more likely to hide their emotions and adjust themselves. Therefore, men are more likely than women to take a negative and indifferent attitude toward their own work and colleagues. In terms of age, older respondents have lower DP levels because safety professionals become more mentally mature as they age and as their ability to cope with their emotions and their interpersonal relationships improve. Therefore, their DP levels decrease with age. In addition, marriage has a positive effect on DP. Married safety professionals have a trusted partner to confide in when they encounter difficulties at work. They are also more likely to receive attention from their partners and can relax mentally and physically. Such an intimate relationship causes them to be enthusiastic regarding things outside of work, leading to a drop in DP levels. In terms of work-related factors, three items had a considerable effect on LPA levels (see Table 3 and Table 9). With an increase in working hours per week, safety professionals’ work enthusiasm is reduced, negative emotions accumulate, the professionals become less and less interested in work, and they tend to exhibit a passive and indifferent attitude toward the people and things around them. Therefore, the longer the weekly work hours, the more serious the DP. Safety professionals gain experience and develop their own personal networks as their working years increase and through promotions. The respondents could get high-level positions because of their hard work, strong leadership, and management skills. They maintain a positive attitude toward their colleagues and work partners and care about things outside of work. Thus, middle and senior managers have lower DP levels than grass-roots managers and ordinary staff. In terms of negative perception factors, safety professionals with relatively high DP levels believe that their social status is not high, society has insufficient recognition of them, their work pressure is high, their career development is limited, their personal expertise and abilities are inadequate, their work environment is not good, and their personality is not suitable for the safety industry (see Table 6 and Table 9).

The above analysis showed that numerous factors have significant effects on EE, LPA, and DP. However, on the whole, only four factors significantly influenced JBL—age, weekly work hours, work experiences, and CSE (see Table 11). The first three factors have been discussed above and will not be explained here. With regard to CSE, the analysis results show that respondents who are CSEs have higher JBL than respondents who are not (see Table 3 and Table 10). In general, CSEs in China assume more safety responsibilities, and their work is more difficult [6]; thus, they require more energy and time, which all lead to easy job burnout. In addition, although the regression analysis results of JBL revealed that only CSE had a significant impact on JBL, Table 6 showed that safety professionals with relatively high JBL believe that their work pressure is high, their interpersonal relationships are complex, their work environment is not good, their personal expertise and abilities are inadequate, and their personality is not suitable for the safety industry (see Table 10).

Due to China’s special national conditions, it is necessary to combine the practical problems in the development of China’s safety industry and discuss the potential impact of 14 specific factors (Q1-1–Q4-3) on the job burnout of safety professionals from the perspective of four negative perceptions of them (see Table 12). In addition, in view of these existing problems, this paper gives relevant policy suggestions from the actual situation as follows:

On the social level, first of all, the social status of safety professionals needs to be improved. All walks of life (especially the government) should attach great importance to the construction of the team of safety professionals, strengthen the study of job burnout of them, and improve their psychological and physiological health level so as to alleviate their job burnout. Secondly, the legal environment should be improved. The government should improve the laws and regulations on work safety, clarify the principal responsibility, safeguard the law in an objective, fair, and just way, and provide a harmonious and orderly legal environment for safety professionals to perform their work. Finally, the news media should correctly guide the public thinking to help them correctly view the occurrence of various accidents, ensure the accuracy, objectivity and fairness of the reports, and change the public’s traditional understanding of safety professionals.

At the professional level, the management model of safety professionals needs to be improved. Relevant departments should provide system and mechanism guarantee for safety practitioners, establish scientific and humanized career development concept, and gradually improve their career development mode. In addition, various ways should be adopted to relieve the working pressure of safety professionals. For example, in terms of work, unnecessary safety checks can be reduced and useless safety information can be cut down. In terms of life, relevant organizations can create rich communication opportunities for safety professionals through recreational activities and other ways in order to ease the tension at work.

At the organizational level, first of all, leaders should pay sufficient attention to safety. They should take the lead in doing a good job in safety work, to build an organizational structure that is convenient for the safety department to handle affairs, actively cooperate with the safety professionals to enhance the safety culture, and improve the status of the safety department in the organization. Secondly, the organization should establish a scientific and objective work assessment and evaluation mechanism. In the work evaluation, through quantitative assessment standards to evaluate the work performance of safety professionals, and as an important basis for promotion, so that safety professionals work and grow in a fair and just environment. Finally, the organization should improve the work treatment of safety professionals, so as to further stimulate their enthusiasm and initiative in work and constantly enhance their sense of achievement and honor.

At the individual level, first of all, individuals need to continue to learn professional knowledge, improve their work abilities, and fully understand the work content of the safety industry. Secondly, on the premise of understanding their own personality, individuals need to develop a positive work attitude, further enhance the ability to adapt to the environment, timely find their own sources of pressure and try to resolve it. Finally, individuals should appropriately adjust their own expectations, actively learn emotional control and stress coping strategies through on-the-job training, take the initiative to seek support and help, and conduct self-assessment and self-mitigation of job burnout.

In addition, this study had several limitations. First, the number of samples was not sufficiently large, and the distribution of subjects was not wide enough; thus, the samples may not represent all conditions of safety professionals. Second, owing to the diversity of factors affecting job burnout, some reasons are not included in the survey; thus, we could not obtain relevant information. Third, the data of the participants were affected by unavoidable factors. For example, many those with severe burnout may be out of work or at home and not picked up during the survey. Fourth, because there are too few studies on burnout of safety professionals at present and the relevant literature is not rich enough, there are still many contents that have not been discussed in this paper. Fifth, on the basis of the data obtained from the questionnaire survey, combined with China’s national conditions, the analysis results were explained using descriptive language; thus, this study was a hypothesis based on the results of data analysis.

## 5. Conclusions

The main contribution of this paper is that it is the first time an investigation on job burnout among safety professionals in various industries (taking China as an example) has been conducted and relevant analyses have been performed. Our results revealed that most safety professionals participating in the questionnaire (98.3%) experienced job burnout, and their job burnout types and related factors were different. These results prove that job burnout is a very serious problem for safety professionals, which also has some connections with their performance in real life. Therefore, the burnout problem of safety professionals must be targeted in future studies. At the same time, more scholars need to pay attention to the research in this area and provide more countermeasures. Despite some limitations, this study is meaningful, which is where the value of this paper lies. Further research must be performed to increase the relevant knowledge in this field so that the actual situation of work stress among safety professionals can be more truly reflected.

## Figures and Tables

**Table 1:** Characteristics of study population and job burnout level (N = 526).

Characteristics		Frequency (*n*)	Percentage (%)
Gender	Male	434	82.5%
	Female	92	17.5%
Age	≤30	153	29.1%
	31–40	195	37.1%
	41–50	143	27.2%
	≥51	35	6.6%
Educational background	Technical secondary school and below	39	7.4%
	Junior college degree	105	20.0%
	Undergraduate degree	282	53.6%
	Graduate degree and above	100	19.0%
Marital status	Unmarried	140	26.6%
	Married	371	70.5%
	Divorced or widowed	15	2.9%
Monthly income (RMB)	≤5000	125	23.7%
	5001–7000	148	28.1%
	7001–10,000	116	22.1%
	≥10,001	137	26.1%
Weekly work hours	≤40	64	12.2%
	41–50	231	43.9%
	51–60	110	20.9%
	61–70	60	11.4%
	71–80	31	5.9%
	≥81	30	5.7%
Work experiences (years)	≤5	169	32.1%
	6–10	143	27.2%
	11–20	143	27.2%
	≥21	71	13.5%
Position level	Ordinary staff	146	27.8%
	Grass-roots managers	220	41.8%
	Middle managers	146	27.8%
	Senior manager	14	2.6%
Certified safety engineer (CSE) or not	Yes	255	48.5%
	No	271	51.5%
Whether the individual held an administrative position	Yes	209	39.7%
	No	317	60.3%
Job burnout level (JBL)	Normal (no burnout)	9	1.7%
	Mild-level burnout	161	30.6%
	Middle-level burnout	306	58.2%
	High-level burnout	50	9.5%

**Table 2:** Evaluation of level of job burnout in the three indicators (N = 526).

Three Indicators of Job Burnout	Percentage (%)	Mean Score	Standard Deviation	Level Evaluation
EE	12.9	16.5	7.738	Normal level
LPA	90.1	12.8	8.726	Burnout level
DP	72.4	13.8	8.504	Burnout level

Note: EE: emotional exhaustion; LPA: lack of personal accomplishment; DP: depersonalization.

**Table 3:** Mean scores of MBI-GS and its subscales according to individual factors and work-related factors (M ± SD).

Variables		EE	LPA	DP	JBL
Gender	Male	16.87 ± 7.68	12.76 ± 8.75	14.14 ± 8.52	1.76 ± 0.65
	Female	14.85 ± 7.83	13.24 ± 8.68	12.09 ± 8.25	1.74 ± 0.61
	t	2.260	−0.480	2.156	0.268
	*p*	0.025	0.632	0.033	0.789
Age	≤30	16.75 ± 7.51	14.94 ± 8.25	16.5 ± 8.64	1.8 ± 0.55
	31–40	16.08 ± 7.38	12.96 ± 8.13	13.24 ± 7.64	1.79 ± 0.63
	41–50	16.77 ± 8.04	10.94 ± 9.34	12.2 ± 8.77	1.67 ± 0.71
	≥51	16.91 ± 9.49	10.83 ± 9.52	11.4 ± 8.78	1.69 ± 0.76
	Pearson correlation coefficient	<0.001	−0.206	−0.213	−0.086
	*p*	0.995	<0.001	<0.001	0.048
Educational background	Technical secondary school and below	18.54 ± 9.25	11.46 ± 9.6	13.46 ± 10	1.74 ± 0.79
	Junior college degree	14.95 ± 8.21	11.85 ± 8.73	11.86 ± 8.33	1.68 ± 0.69
	Undergraduate degree	17.07 ± 7.16	13.16 ± 8.53	14.57 ± 8.34	1.78 ± 0.61
	Graduate degree and above	15.81 ± 7.92	13.54 ± 8.92	13.71 ± 8.31	1.78 ± 0.61
	Pearson correlation coefficient	−0.019	0.076	0.06	0.043
	*p*	0.657	0.083	0.166	0.33
Marital status	Unmarried	17.29 ± 8.12	15.06 ± 8.84 ^a+^	16.90 ± 8.99 ^a+^	1.83 ± 0.60
	Married	16.14 ± 7.47	12.01 ± 8.40 ^a−^	12.59 ± 7.83 ^a−^	1.73 ± 0.65
	Divorced or widowed	18.60 ± 10.13	12.93 ± 12.15	14.20 ± 12.47	1.8 ± 0.68
	F	1.672	6.343	13.722	1.368
	*p*	0.189	0.002	<0.001	0.256
Monthly income (RMB)	≤5000	17.19 ± 8.35	14.18 ± 9.35	13.39 ± 9.43	1.72 ± 0.71
	5001–7000	17.06 ± 7.83	12.70 ± 8.85	15.51 ± 8.63	1.80 ± 0.64
	7001–10,000	15.88 ± 7.53	13.82 ± 8.80	13.55 ± 7.96	1.73 ± 0.58
	≥10,001	15.85 ± 7.20	10.95 ± 7.62	12.46 ± 7.67	1.75 ± 0.63
	Pearson correlation coefficient	−0.083	−0.099	−0.052	−0.007
	*p*	0.058	0.023	0.233	0.869
Weekly work hours	≤40	13.58 ± 8.31	12.11 ± 8.94	13.22 ± 8.72	1.75 ± 0.62
	41–50	15.37 ± 7.45	13.29 ± 8.47	12.93 ± 8.25	1.68 ± 0.65
	51–60	17.94 ± 6.82	12 ± 8.44	14.47 ± 8.15	1.81 ± 0.57
	61–70	16.5 ± 7.5	11.72 ± 8.74	11.67 ± 7.47	1.7 ± 0.67
	71–80	20.48 ± 7.79	12.39 ± 8.38	17.1 ± 9.35	1.97 ± 0.66
	≥81	22.33 ± 7.3	16.8 ± 10.74	19.8 ± 9.16	2 ± 0.74
	Pearson correlation coefficient	0.265	0.042	0.152	0.111
	*p*	<0.001	0.338	<0.001	0.011
Work experiences (years)	≤5	16.83 ± 7.93	14.44 ± 8.63	15.99 ± 9.04	1.81 ± 0.57
	6–10	16.01 ± 7.26	12.81 ± 8.19	13.75 ± 8.16	1.77 ± 0.65
	11–20	16.62 ± 7.3	12.08 ± 8.45	12.36 ± 7.77	1.71 ± 0.68
	≥21	16.58 ± 9.08	10.65 ± 9.99	11.45 ± 8.21	1.68 ± 0.71
	Pearson correlation coefficient	−0.019	−0.16	−0.196	−0.087
	*p*	0.668	<0.001	<0.001	0.045
Position level	Ordinary Staff	16.58 ± 8.37	14.20 ± 8.91	14.17 ± 8.72	1.77 ± 0.64
	Grass-roots managers	16.74 ± 7.32	12.99 ± 8.56	14.64 ± 8.66	1.75 ± 0.63
	Middle managers	15.84 ± 7.50	11.51 ± 8.37	12.34 ± 7.81	1.73 ± 0.64
	Senior manager	19.43 ± 9.56	10.29 ± 11.21	11.36 ± 9.30	1.86 ± 0.86
	Pearson correlation coefficient	−0.018	−0.126	−0.088	−0.023
	*p*	0.68	0.004	0.044	0.602
Certified safety engineer (CSE) or not	Yes	16.75 ± 7.12	12.58 ± 8.28	14.03 ± 7.68	1.82 ± 0.62
	No	16.3 ± 8.29	13.09 ± 9.14	13.55 ± 9.22	1.70 ± 0.66
	t	0.655	−0.674	0.643	2.124
	*p*	0.513	0.501	0.52	0.034
Holds an administrative position or not	Yes	16.46 ± 7.67	11.81 ± 8.66	13.34 ± 8.27	1.75 ± 0.64
	No	16.55 ± 7.79	13.52 ± 8.72	14.07 ± 8.66	1.76 ± 0.64
	t	−0.128	−2.211	−0.976	−0.1034
	*p*	0.898	0.028	0.329	0.918

Note: Values are presented as mean ± standard deviation. “a” means that there is a significant difference between the two groups of samples; the plus and minus signs denote size; “+” means significantly large, and “−” means significantly small. EE: emotional exhaustion; LPA: lack of personal accomplishment; DP: depersonalization; JBL: job burnout level.

**Table 4:** Selection and scores of negative perception questions (N = 526).

Dimension of Negative Perception	Options	Percentage (%)
Negative perception of social factors (Q1)	The social status of safety professionals is not high (Q1-1)	80.4
	The social cognition and identification of safety professionals is insufficient (Q1-2)	79.8
	Many aspects of the social safety responsibility system are not perfect, and the implementation is not enough (Q1-3)	78.1
Negative perception of occupational factors (Q2)	Safety professionals are under great pressure (Q2-1)	82.5
	Safety professionals face complex relationships (Q2-2)	70.7
	Safety professionals have limited career development (Q2-3)	79.7
	The working objects of safety professionals vary greatly (Q2-4)	67.9
Negative perception of organizational factors (Q3)	The working environment is not good (Q3-1)	39.7
	Leaders pay insufficient attention to safety (Q3-2)	60.8
	The organizational structure is not friendly to the safety department (Q3-3)	70.2
	The pay for safety work is lower (Q3-4)	56.7
Negative perception of personal factors (Q4)	Lack of professional knowledge and ability (Q4-1)	58.0
	The personality is not suitable for working in the safety industry (Q4-2)	45.4
	Personal awareness of the safety industry is insufficient, and it is difficult to achieve self-value (Q4-3)	56.7

**Table 5:** Scores of negative perception questions.

Dimension of Negative Perception	Score	Frequency (*n*)	Percentage (%)
Negative perception of social factors (Q1)	1	86	16.3
	2	152	28.9
	3	288	54.8
Negative perception of occupational factors (Q2)	1	41	7.8
	2	128	24.3
	3	143	27.2
	4	214	40.7
Negative perception of organizational factors (Q3)	1	140	26.6
	2	178	33.8
	3	132	25.1
	4	76	14.4
Negative perception of personal factors (Q4)	1	284	54.0
	2	168	31.9
	3	74	14.1

Note: The four dimensions of negative perception are scored based on the number of options selected in these questions, that is, one option is scored as 1, two options as 2, and so on.

**Table 6:** Test and analysis results of negative perception and job burnout (N = 526).

Options Code	EE	LPA	DP	JBL
Q1-1	1.103	2.849 **	2.351 *	−0.043
Q1-2	1.815	−0.559	2.217 *	1.192
Q1-3	1.585	0.873	0.412	0.597
Q2-1	3.678 **	0.058	2.122 *	2.436 *
Q2-2	3.511 **	0.748	1.367	2.156 *
Q2-3	1.658	0.166	2.311 *	1.143
Q2-4	2.694 **	0.897	0.991	1.128
Q3-1	4.667 **	3.115 **	4.007 **	2.837 **
Q3-2	−0.143	0.999	1.735	0.619
Q3-3	1.034	0.433	1.287	0.811
Q3-4	2.342 *	−0.127	1.781	0.698
Q4-1	−4.042	1.115	−3.565	−2.349 *
Q4-2	2.78 **	3.925 **	5.857 **	2.281 *
Q4-3	0.672	2.177 *	0.77	0.012
Q1	0.128 **	0.066	0.109 *	0.044
Q2	0.225 **	0.041	0.119 **	0.113 **
Q3	0.161 **	0.082	0.185 **	0.119 **
Q4	−0.018	0.214 **	0.089 *	0.011

Note: The values from Q1-1 to Q4-3 in the table are t-values. T > 0 indicates that the respondents who chose this option have higher scores of job burnout than those who did not. The values from Q1 to Q4 in the table are Pearson correlation coefficients. A value greater than 0 indicates that the respondents who chose this option have a higher job burnout than those who do not. The symbol “*” represents a significant difference, with “*” representing *p* < 0.05 and “**” representing *p* < 0.01.

**Table 7:** Results of regression analysis of EE.

Variable	B	Beta	*p*	Variance Inflation Factor (VIF)
(Constant)	9.373		<0.001	
Gender	−0.552	−0.027	0.525	1.058
Weekly work hours	1.353	0.229	<0.001	1.071
Q1	−0.159	−0.015	0.755	1.426
Q2	1.168	0.148	0.003	1.486
Q3	0.741	0.097	0.047	1.381
Q4	−0.441	−0.041	0.331	1.044

Note: Adjusted R-Square = 0.1; VIF < 5.

**Table 8:** Results of regression analysis of LPA.

Variable	B	Beta	*p*	Variance Inflation Factor (VIF)
(Constant)	10.148		<0.001	
Age	−1.262	−0.131	0.037	2.198
Weekly work hours	0.402	0.060	0.166	1.062
Work experiences (years)	−0.070	−0.008	0.893	2.189
Position level	1.099	1.099	1.099	1.099
Whether to hold an administrative position	1.099	0.062	0.185	1.213
Monthly income (RMB)	−0.530	−0.068	0.152	1.254
Q1	0.388	0.033	0.511	1.450
Q2	−0.427	−0.048	0.354	1.509
Q3	0.553	0.064	0.208	1.453
Q4	2.055	0.170	<0.001	1.123

Note: Adjusted R-Square = 0.067; VIF < 5.

**Table 9:** Results of regression analysis of DP.

Variable	B	Beta	*p*	Variance Inflation Factor (VIF)
(Constant)	15.803		<0.001	
Gender	−1.981	−0.089	0.042	1.100
Age	−1.085	−0.115	0.058	2.140
Work experiences (years)	−0.901	−0.110	0.069	2.147
Position level	−0.736	−0.070	0.120	1.183
Weekly work hours	0.818	0.126	0.004	1.094
Q1	0.028	0.002	0.960	1.446
Q2	0.060	0.007	0.892	1.513
Q3	1.507	0.179	<0.001	1.421
Q4	0.132	0.011	0.799	1.120

Note: Adjusted R-Square = 0.098; VIF < 5.

**Table 10:** Results of regression analysis of OBL.

Variable	B	Beta	*p*	Variance Inflation Factor (VIF)
(Constant)	1.849		<0.001	
Age	−0.032	−0.045	0.472	2.150
Work experiences (years)	−0.044	−0.071	0.264	2.197
Certified safety engineer (CSE) or not	−0.134	−0.105	0.019	1.080
Weekly work hours	0.040	0.083	0.059	1.037
Q1	−0.051	−0.060	0.246	1.445
Q2	0.061	0.094	0.073	1.484
Q3	0.062	0.098	0.056	1.418
Q4	−0.025	−0.028	0.541	1.115

Note: Adjusted R-Square = 0.32; VIF < 5.

**Table 11:** Effects of the ten demographic variables and four negative perception factors on job burnout.

Demographic Variables and Four Negative Perception Factors	EE	LPA	DP	JBL
Gender	◯		◯	
Age		◯	◯	◯
Educational background				
Marital status		◯	◯	
Monthly income (RMB)		◯		
Weekly work hours	◯		◯	◯
Work experience (years)		◯	◯	◯
Position level		◯	◯	
Certified safety engineer (CSE) or not				◯
Whether the individual held an administrative position		◯		
Q1-1		◯	◯	
Q1-2			◯	
Q1-3				
Q2-1	◯		◯	◯
Q2-2	◯			◯
Q2-3			◯	
Q2-4				
Q3-1	◯	◯	◯	◯
Q3-2				
Q3-3				
Q3-4	◯			
Q4-1		◯	◯	◯
Q4-2	◯		◯	◯
Q4-3		◯		

Note: The symbol “◯” means that the independent variable has a significant influence on the dependent variable. EE: emotional exhaustion; LPA: lack of personal accomplishment; DP: depersonalization; JBL: job burnout level.

**Table 12:** The potential impact of 14 specific factors on the job burnout of safety professionals from the perspective of four negative perceptions.

Dimensions of Negative Perception	Specific Factors	The Actual Potential Impact on Job Burnout for Safety Professionals
Q1	Q1-1	Grassroots safety professionals are at the end of law enforcement behavior and the end of the administrative system, and are often the most obvious ones responsible in accident liability investigation. The grassroots safety professionals are of lower status. They have to shoulder the responsibilities for accidents so they are often under great pressure.
	Q1-2	Because the most direct goal of safe work is to “prevent and reduce accidents”, namely a large amount of the work is preventive work. There is no obvious causal relationship between “safe production work” and “do not happen accidents”. Therefore, when there is no accident, people often do not realize the labor of the vast number of safety professionals and the huge potential value. However, when an accident happens, the authorities, the news media, the families of the accident victims, and the public may all blame the safety professionals. Facing this situation, the safety professionals must prove that they are not responsible for the accident. Thus, during work, they always suffer burnout for fear of accidents.
	Q1-3	The accountability system is not objective and not standard, which is the most important factor that leads to the instability of the team of safety professionals. In fact, some problems in the existing conditions, is an ordinary grassroots safety professionals can not solve. The scope and conditions of exemption from liability of safety professionals are often difficult to be reflected in the accountability process. This has caused great psychological and ideological pressure to safety professionals and they are full of complaints.
Q2	Q2-1	Safety professionals face frequent safety checks at all levels, numerous safety data and documents, and heavy safety affairs. At the same time, they bear the greater safety responsibility and work pressure. In addition, some safety professionals have multiple roles, although belonging to the safety department, but have to complete the tasks of other departments, which leads to a very limited time to seriously devote to safety work.
	Q2-2	Some employees ignore the requirements of safety professionals, and some even do not obey the rules and refuse safety check. Some leaders intervene safety work, command concerned departments to change penalty decision at will. Faced with complex interpersonal relationships, safety professionals are often at a loss to deal with problems such as violations of laws and regulations and hidden dangers.
	Q2-3	Ordinary safety professionals have limited space for growth and progress, and it is hard for them to get promoted. Their work motivation and initiative has been severely damaged. For example, even the leaders in charge of work safety in local governments are often low in prestige, seniority, and status, and their efforts to coordinate and promote work are not strong. This makes some safety professionals lack confidence in their career.
	Q2-4	Safety professionals need to report safety work to the leadership, carry out safety management for ordinary employees, and effectively communicate with various departments to seek cooperation in safety. This puts forward a higher requirement for the personal ability of safety practitioners, which leads to job burnout of some safety professionals.
Q3	Q3-1	Some safety professionals need to carry out related safety management work in the harsh environment of high-risk industries all year round. They work in such a high-pressure environment all year round, which requires not only a strong psychological adjustment ability but also the necessary health costs. Their physical and mental conditions are in an unhealthy state.
	Q3-2	Many leaders pay attention to safety orally, but their investment in safety is very little, and they do not care about the training of safety professionals teams and the deployment of safety work. Individual leaders are not concerned about violating rules or regulations punishment, and after causing a safety accident, even still do not know how to repent. Some safety professionals lost the support and help of the leadership completely in the process of implementing various work tasks.
	Q3-3	Production department, equipment department, and other relevant functional departments do not have a deep understanding of the concept of “one post, two responsibilities”, they think that safety checks, hidden danger rectification, safety training, violation correction, and so on should be the matter of the safety department and safety professionals. They turn a blind eye to and avoid the safety work in their area of responsibility. This makes it difficult for safety professionals to communicate and cooperate with other departments.
	Q3-4	Safety professionals do not receive recognition from others and material rewards that are deserved in the process of work. This may be because the organization has problems in the way of safety work performance assessment, or the organization does not have a scientific and objective evaluation system.
Q4	Q4-1	Due to some reasons for the development of the safety industry, there are still many safety workers who do not graduate from safety-related majors, and they are insufficient in professional knowledge and ability. In addition, safety professionals have no time to continue learning and training because of heavy work tasks, and their comprehensive ability to improve is slow. This makes it difficult for them to handle complex safety work.
	Q4-2	There are many types of personality that are not suitable for safety management. For example, a very strong sense of self-responsibility, which mainly refers to the work within any scope of responsibility, show excessive attention and invest too much energy, and once the degree of attention and input surpass a certain level, this will lead to job burnout. Other personality traits are not listed here.
	Q4-3	People who are prone to job burnout show idealism and perfectionism and have a desire for higher achievement. Some safety professionals expect too much of themselves. They tend to set goals beyond their ability at work, and then pursue the goal with full energy and even extraordinary efforts. However, they often fail to achieve their self-worth because their goals are impractical.

## Data Availability

Data can be available upon request to the authors.
